# Patient Perspectives to Inform a Health Literacy Educational Program: A Systematic Review and Thematic Synthesis of Qualitative Studies

**DOI:** 10.3390/ijerph16214300

**Published:** 2019-11-05

**Authors:** Margot Jager, Janine de Zeeuw, Janne Tullius, Roberta Papa, Cinzia Giammarchi, Amanda Whittal, Andrea F. de Winter

**Affiliations:** 1 Department of Health Sciences, University Medical Center Groningen and University of Groningen, 9700 AD Groningen, The Netherlands; j.m.tullius@umcg.nl (J.T.); a.f.de.winter@umcg.nl (A.F.d.W.); 2 Department of Medical Sciences, Educational Institute, University Medical Center Groningen, University of Groningen, 9700 RB Groningen, The Netherlands; 3 Regional Health Agency Marche Region, 60125 Ancona, Italy; roberta.papa@regione.marche.it (R.P.); cinzia.giammarchi@regione.marche.it (C.G.); 4 IRCCS INRCA, 60124 Ancona, Italy; 5 Department of Psychology & Methods, Jacobs University, 28759 Bremen, Germany; a.whittal@jacobs-university.de

**Keywords:** health literacy, patient-centeredness, patient perspectives, education, learning outcomes, qualitative research

## Abstract

Patient-centred care is tailored to the needs of patients and is necessary for better health outcomes, especially for individuals with limited health literacy (LHL). However, its implementation remains challenging. The key to effectively address patient-centred care is to include perspectives of patients with LHL within the curricula of (future) healthcare providers (HCP). This systematic review aimed to explore and synthesize evidence on the needs, experiences and preferences of patients with LHL and to inform an existing educational framework. We searched three databases: PsychInfo, Medline and Cinahl, and extracted 798 articles. One-hundred and three articles met the inclusion criteria. After data extraction and thematic synthesis, key themes were identified. Patients with LHL and chronic diseases encounter multiple problems in the care process, which are often related to a lack of person-centeredness. Patient perspectives were categorized into four key themes: (1) Support system; (2) Patient self-management; (3) Capacities of HCPs; (4) Barriers in healthcare systems. “Cultural sensitivity” and “eHealth” were identified as recurring themes. A set of learning outcomes for (future) HCPs was developed based on our findings. The perspectives of patients with LHL provided valuable input for a comprehensive and person-centred educational framework that can enhance the relevance and quality of education for (future) HCPs, and contribute to better person-centred care for patients with LHL.

## 1. Introduction

Effective person-centred prevention and care is embraced by many healthcare organizations given the rising number and impact of chronic diseases. Unfortunately, people with limited health literacy (LHL) do not benefit sufficiently from health services due to a lack of tailoring to their capacities and needs. People with LHL can be defined as persons who have difficulty in accessing and understanding health information, and in appraising and applying such information in making decisions related to health and healthcare [1,2]. LHL is associated with faster disease progression, a higher prevalence of co-morbidities and more psychological problems [3,4,5]. These poorer health outcomes may be partially due to patients with LHL often being less able to discuss their needs, and with more difficulties with changing or maintaining health behaviours and managing their illness and treatment [6,7].

To mitigate health literacy-related problems, person-centred prevention and care have been advocated, as these are associated with improved self-management, patient satisfaction, adherence and health outcomes [8,9,10]. In person-centred care, individuals, families and communities are served by and are able to participate in trusted health systems that respond to their needs in humane and holistic ways [11]. To be able to provide person-centred care to these vulnerable patients, (future) healthcare providers (HCPs) should be trained in knowledge, skills and attitude towards health literacy as this may improve healthcare outcomes of patients with LHL.

Already in 2004, the US Institute of Medicine advised incorporating health literacy into the curricula of (future) HCPs to promote person-centred care [12]. Nevertheless, HCPs in the European Union have hardly been trained on health literacy competencies and often lack the necessary skills to address LHL in patients effectively [5,13]. Despite scarce evidence regarding the effectiveness of health literacy (HL) education in curricula, the existing research shows that after training, HCPs are more aware of the needs of patients with LHL, and are more skilled in providing comprehensible information, enabling shared decision-making and promoting self-management [14,15]. In addition, HL education for undergraduate HCPs improves knowledge of health literacy and skills in communicating clear information to patients with LHL [16].

**Figure 1:**
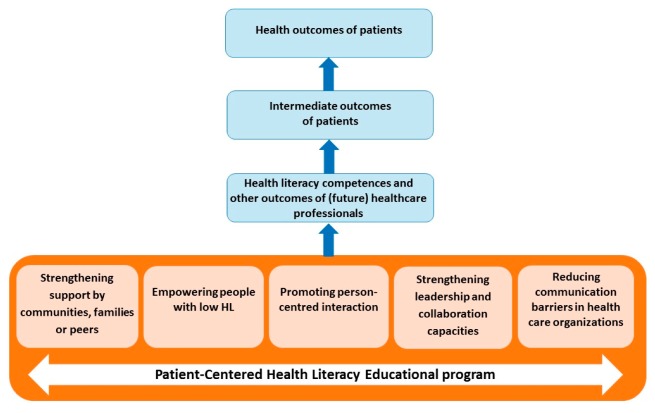
Comprehensive health literacy educational framework promoting person-centred care.

To successfully improve future HCPs capacities, a comprehensive, evidence-informed HL educational program is recommended [14,17]. In the context of the IMPACCT (IMproving PAtient-centered Communication Competences) project (http://healthliteracycentre.eu/impacct/), a preliminary educational framework (see Figure 1) was developed based on a comprehensive health literacy intervention model [7]. The main objective of IMPACCT is to improve the relevance and quality of education of medical and nursing students in Europe through the development, implementation, evaluation and dissemination of an evidence-based health literacy educational program. The framework presented in Figure 1 entails five core educational domains, which should be covered in training to increase the abilities of students and ensure person-centred prevention and care for patients with LHL.

The key to effectively addressing LHL within our educational model is to include patient perspectives. A better understanding and integration of patients’ needs, experiences, and preferences contributes to increased practice-oriented education and consequently, to true person-centred care. Therefore, our first aim was to explore and synthesize available evidence on the needs, experiences and preferences of patients with LHL and chronic diseases. Our second aim was to formulate learning outcomes based on patient perspectives and to inform the educational framework.

## 2. Methods

This study was conducted within the framework of the IMPACCT project (http://healthliteracycentre.eu/impacct/). IMPACCT aims to improve the relevance and quality of education of medical and nursing students in Europe through the development and implementation of an evidence-informed Health Literacy Educational Program (HL-EP).

### 2.1. Study Design

To be able to understand patients’ needs, experiences and preferences, we conducted a systematic review of qualitative studies [18,19]. We chose to include only qualitative studies because in these the patient’s experiences and perspectives are well captured since detailed descriptions and vivid quotations are included. 

### 2.2. Search Strategy

A comprehensive search strategy was developed by an information specialist using a combination of subject headings (MeSH terms) and free-text terms to identify relevant articles related to our study objectives. The searches using these headings and terms were conducted in PsychInfo, Medline and Cinahl without time constraints until the 13th of February 2018. The selected combination of key words resulted in the following search strategy:

(MH “Communication Barriers+” OR TI (communication barriers OR qualitative OR focus group*) OR AB (communication barriers OR qualitative OR focus group*)) AND (TI (patient OR patients) OR AB (patient OR patients)) OR (TI (patient OR patients) OR AB (patient OR patients )) N3 (TI (satisfaction OR perspective* OR experience*) OR AB (satisfaction OR perspective* OR experience*)) AND (MH “Health literacy” OR TI health literacy OR AB health literacy).

### 2.3. Selection of Articles

After completion of the search and removal of duplicates, the titles and abstracts were screened for potential eligibility for inclusion in the systematic review. Four reviewers (A.W., M.J., R.P., C.G.) conducted an abstract and full text review of potentially eligible articles from the title screening, to assess whether the selected articles met the inclusion criteria. Any disagreements about article inclusion were resolved by discussion in the reviewer group to reach consensus.

Articles were included if:

- They provided information on perspectives or experiences related to healthcare of adult participants (18+) with a chronic disease and LHL, described with qualitative research. Participants of all studies will be hereinafter referred to as ‘patients’. We identified patients with LHL as persons who have difficulty in accessing and understanding health information, and in appraising and applying such information in making decisions related to health and healthcare [1].
- Studies were conducted in developed countries (USA, Europe, New Zealand, or Australia).
- They were written in English, Italian, German or Dutch, as these languages were spoken within the research team.

Articles were excluded if:

- Experiences of healthy persons with LHL in the general population were described (e.g., articles on screening in the general population).
- They focused on HL research among children or adolescents; articles including participants with a large age range were not included if they did not describe results for adult patients specifically.
- They were research related articles (e.g., articles describing how persons with LHL struggle with informed consent within research or how HL impacts participation in research).
- They focused on perspectives of HCPs on patients with LHL.

### 2.4. Data-Extraction and Analysis (Phase I)

The aim of data extraction in phase I was to identify basic study characteristics (i.e., study design, sample size, research questions, etc.) and the needs, experiences and preferences of patients with LHL and chronic diseases. In addition, quotes illustrating patient perspectives particularly effectively and clearly were extracted.

Prior to data extraction, five articles were reviewed by all members as a pilot to ensure common strategy and understanding, and to promote the quality of data extraction. Data were extracted from articles, which met all the criteria, divided among four reviewers (A.W., M.J., R.P., C.G.). Regular Skype meetings were held to discuss the extraction process. These discussions were used to check for accuracy in extraction and resolve any disagreements or uncertainties. Altogether, the extracted data, included in a devoted Excel sheet, comprised the following information:

- “Descriptives”, such as country of study, language of the article, publication year, sample size, setting, study design, objectives or research questions, methods, topic area, target population, HL indicator (if used), type and stage of chronic disease, and type of HCPs (if applicable).
- “Content”, such as key concepts/themes identified, main experiences/ideas of patients, facilitating factors, barriers, quotations for illustration of patients and others, recommendations for healthcare.

The aim of analysis in phase I was to identify key themes in the data. All reviewers independently analyzed the data of their assigned articles and formulated key themes. These themes were compared, adapted and specified until consensus was reached.

### 2.5. Data-Extraction and Analysis (Phase II)

The aim of data extraction and analysis in phase II was to identify all themes formulated in phase I per article systematically. Explicit author interpretation for identifying all themes per article, a strategy known as a ‘semantic approach’, was used, i.e., themes were identified within the explicit written meanings of the information, without further interpretation of what was said or written [20]. The data extraction Excel sheet from phase I was used, and when clarification was needed, the original article was checked. Categorization of the articles per theme was discussed for each article, when necessary. Finally, we checked whether the themes fit within the domains of our provisional HL educational framework. The HL education framework includes the following five main educational domains, with which the themes from the articles in phase I were aligned:

- Support system: defined as the social network of communities, families or peers supporting the patient with LHL.
- Patient empowerment: defined as the inherent capacity to be responsible for maintaining and promoting one’s own health.
- Patient–provider interaction: defined as verbal and non-verbal communication exchanges between HCPs and patients with LHL, as well as everything that might influence the interaction between the patient and the HCP (e.g., perceived time, respect).
- Leadership and collaboration: defined as competencies and actions initiated by a HCP in order to accommodate the patient with LHL (e.g., putting HL on the agenda, interaction between HCPs, and coordination of care).
- Communication barriers: defined as obstacles within the healthcare system that appear to be a barrier for patients with LHL (e.g., written materials, hospital navigation, front desks, hospital websites).

To ensure reliability, two researchers (JdZ and JT) reviewed themes identified from phase I, and categorized articles per theme. As it was often difficult to clearly distinguish themes and subthemes from one another, in the case of disagreement, two other researchers (M.J., A.d.W.) were consulted. The process and outcomes from phase II were discussed with all authors to ensure agreement. 

### 2.6. Development of Learning Outcomes

Based on the final themes that resulted from the analysis, a set of learning outcomes was formulated. Learning outcomes represent key issues to be developed during training and to be practiced at work. Specifically, the key issues highlight behaviours promoting positive health outcomes for patients with LHL and contrast attitudes creating barriers towards an effective healthcare process. To develop the learning outcomes, we used Bloom’s Taxonomy of Educational Objectives [21] and advice of educational specialists. The learning outcomes were developed during four brainstorm sessions (by M.J., J.d.Z., J.T., A.d.W.).

## 3. Results

**Figure 2:**
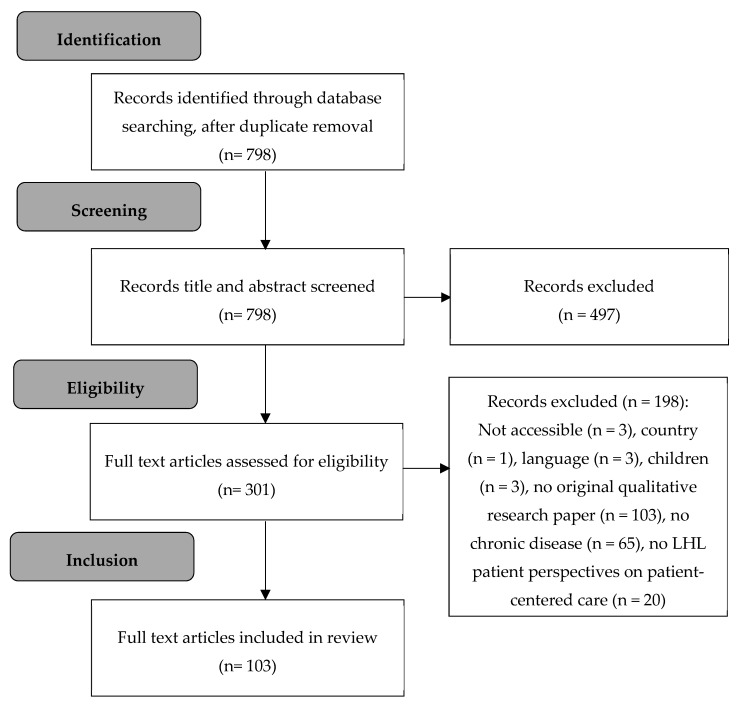
Flow chart of study selection.

The search of online databases yielded 798 articles, after duplicates were removed. Based on the initial screening of title and abstract, 497 articles were excluded as they did not meet the inclusion criteria. The remaining 301 articles were obtained in full text if possible and assessed for eligibility. Subsequently, 198 articles were excluded, as inclusion criteria were not met. Ultimately, 103 articles were retained for the analysis (Figure 2).

### 3.1. Descriptives

The main characteristics of the studies are summarized in Table 1. The majority of the studies were conducted in the USA, UK and Australia (77.9%), used a variety of qualitative methods, and included patients with different types of chronic diseases. A minority of the included studies used specific tools (e.g., scales, questionnaires, etc.) to assess the level of HL (29.9 %). Most studies used proxies of LHL to describe their participants, such as low socioeconomic status or cultural background (immigrants, language problems). 

The studies included a total of 3628 participants, with an age range of 18–85. Several studies included participants with a diverse cultural and ethnic background (34%), while 59.1% of the included participants were female.

### 3.2. Themes

Patients identified different aspects that they viewed as relevant to their care process, all of which fit under the umbrella theme “person-centred care”. The analysis of patient’s perspectives revealed four themes: support system, patient self-management, HCPs interpersonal capacities, and barriers in health care system as displayed in Table 2. These themes can all be related to the domains described in the educational framework (Figure 1). In addition, two recurring themes were identified: “Cultural sensitivity” and “eHealth”. These themes were mentioned by patients in relation to all four key themes. Patients’ positive as well as negative experiences seemed to be related to the extent to which their expectations and preferences were met. They emphasized the importance of being treated as a person with specific needs that should be taken into account by health organizations and professionals. 

Based on the analysis of the included articles, sub-themes were identified. The sub-themes do not entail a hierarchical order. Table 3 shows an overview of themes and sub-themes illustrated with a patient quotation.

### 3.3. Support System

Several articles mentioned the importance of combining multiple sources of support in the care process. As indicated by patients with LHL, having multiple sources in a dedicated support system consisting of family, friends, peers, and their physician is necessary to sustain motivation over a longer period of time. For example, one patient with diabetes described the significant level of support she received from her adult daughter and son (e.g., how they encouraged her to exercise), the support she had from co-workers (e.g., exchanging healthy recipes) as well as how her physician had motivated her. Therefore, one factor may inspire a patient to adhere, but multiple factors may be instrumental in sustaining that motivation long-term. The different sources of support are described in the following in more detail.

#### 3.3.1. Family and Friends

Patients with LHL with chronic conditions often felt emotionally supported by their family. They valued their time with family members and this often served as a motivation cue for patients to manage their condition or continue treatment [23,119]. In addition, patients with LHL reported that family members are supportive in daily life activities (e.g., accompanying them on walks, buying exercise equipment, medications and dietary changes) which can facilitate establishing independence and confidence [22,23,24].

According to patients, family was also an important source of information. Especially family members with personal disease experience were particularly supportive and instrumental [38,119]. In contrast, patients reported family members might play a negative role in the care process (e.g., spouses whom are less supportive or counterproductive) [23]. A patient explained: “*Not at all…especially my husband. He does not play any role [in supporting me]. Not like he don’t love me…he does…by I guess he does not know the outcome maybe [laughing]*” [23].

Furthermore, patients perceived the presence of a family member during consultations as a facilitator in the communication process. They brought along family members or friends to doctor’s appointments for emotional support and to help them understand information and ask questions [24,30,68]. For patients who do not speak the native language, family members sometimes served as informal interpreters in the communication with HCPs.

#### 3.3.2. Peer Contact

Patients described how other patients are a strong source of emotional and social support. In some studies, participants articulated the wish to formalize such peer support relationships early on when they start severe treatment (such as hemodialysis), so that they could learn “the ropes from someone who’s been through this” [24].

Patients with LHL also articulated that social peer support groups facilitated their understanding of their own condition, other patients’ experiences and learning about the disease and treatment outcomes. It was pointed out that peer support groups serve as places of reassurance and understanding which doctors often do not provide [24,49,56,74,94].

#### 3.3.3. Religion and Spirituality

Religion and spirituality were mentioned by patients with LHL to be part of their support system. Patients reported that their faith played a big role in dealing with the diagnosis of a chronic disease and carried them through the hardships of their illness. Praying to a higher spirit and seeing their disease as part of a bigger plan was seen as a source of hope and strength by patients.

#### 3.3.4. Healthcare Provider Support

Patients with LHL point out the importance of feeling supported by their HCP as a motivating cue and facilitator of self-management and independence. Showing concern for and true interest in the patient is valued by patients [22,23,35,41,48,59,60] while HCP support is mentioned to be especially important to female patients describing their physician as “*genuinely caring.. [and that] she encourages me*” [23].

### 3.4. Patient Self-Management

According to patients with LHL, self-management of their chronic disease can be supported by perceiving autonomy and control, gaining knowledge, finding motivators to change and maintain behaviour, and monitoring by HCPs.

#### 3.4.1. Autonomy and Control

Patients with LHL want to feel personally responsible for their own health and in control of their care process and disease management. Patients with LHL often perceived a lack of control regarding different aspects of disease management. Personal autonomy when making choices concerning one’s own health or when discussing information and consent is desired by patients. Feeling in control and autonomous provides patients a sense of comfort. However, for many patients, taking control over their own health takes time and guidance. Patients indicated that they first needed to accept their situation. After acceptance they realized that they needed to take charge of their own health and care process [22,25,31,46,61,64,66,72,77,80,84,102].

#### 3.4.2. Gaining Knowledge

Patients with LHL expressed the relevance of knowledge about their disease, symptoms, and results to feel more in control and confident about managing their condition. By having more knowledge of their own disease, patients felt like they are regaining power and autonomy. The perceived need for patients to acquire more knowledge about their condition might influence the extent to which patients actively seek knowledge themselves [44,72,80]. A patient explains: “*Being educated on how to educate yourself would be a lifesaver”* [38].

#### 3.4.3. Motivators

Patients with LHL and chronic diseases often need to change their behaviour to take control over their disease. Some patients emphasized the gap between knowing and doing, which was an on-going struggle for them. For these patients, important motivators were their family members. They wanted to be a good example to their children and stay healthy to be able to see their (grand)children grow up [23,119]. From a fear perspective, problems or deaths of family members or close friends suffering from chronic diseases, served as motivators to change health behaviours. Actually seeing results, such as losing weight, was another motivating cue mentioned. If a HCP’s advice was perceived as unreasonable and not doable, patients reported difficulties with adhering to lifestyle changes.

#### 3.4.4. Monitoring

According to patients with LHL, being held accountable and monitored by their HCPs resulted in positive behaviour change and increased self-management [70,79,123]. It was mentioned in relation to the use of digital self-management tools that HCP involvement and feedback is crucial as a form of positive reinforcement for patients to keep using the health application. To stay motivated, it seems essential to know that the doctor is connected to the application [34,80,89].

### 3.5. HCPs Interpersonal Capacities

Patients with LHL described their perspectives on patient–provider relationships and what they think are important skills HCPs should have. Relevant HCPs capacities concerned the ability to show respect and understanding, to use a comprehensible communication style, and to involve patients in decision making according to their needs.

#### 3.5.1. Respect and Understanding

Patients with LHL indicated the importance of being seen as a person and feeling understood and respected as a requirement for a trustworthy and good relationship with their HCP. Such a relationship enabled patients to understand information, to feel free to ask questions, and to adhere to their providers’ advice and recommendations [48,59,65,66,72,76,77,87,88,96,99].

Patients with LHL pointed out the importance of feeling supported by their HCP as a motivating cue and facilitator of self-management and independence [79,123]. Showing concern for and true interest in the patient was valued by patients. A patient explains: “*I didn’t feel like they were really interested. They were just talking… I just want my doctor to recognize who I am.. and they say: well let’s see how you doing”* [72].

#### 3.5.2. Comprehensible Communication Style

According to patients with LHL, HCPs tend to use medical jargon leading to misunderstanding and non-understanding of medical information, whereas patients preferred easy language and less use of medical jargon [29,30,73,101]. Patients perceived the use of medical jargon as lack of communication, which had a negative impact on their health, self-management behaviours, emotions, and mental well-being [57,92,106]. A patient explains: *“[The doctor] was rattling off all these things that I needed to do…and my brain just shuts off. It was overload”* [91].

In addition, HCPs should be mindful about the amount of information they provide to patients with LHL [34,38,49,83,91,98] and align information on diagnosis and treatment with patient’s needs [43,79]. Many patients mentioned that they prefer getting a diagnosis and treatment information separately.

#### 3.5.3. Decision Making

Patients with LHL mentioned that when they receive information by HCPs in an understandable way, and when they are engaged in decision-making about their own care, it results in better self-efficacy, self-management, and adherence to treatment [70,79,123]. However, patients reported barriers to asking questions because of time constraints during encounters with HCPs [45,47,50,68,94]. Due to limited time, from the perspectives of patients with LHL, doctors do not use shared decision making or discuss all treatment options [56,80]. In addition, patients report they avoid asking questions also because they do not know or are unsure on what to ask and/or they feel embarrassed to display a lack of understanding.

### 3.6. Barriers in the Healthcare System

Patients perceived the incomprehensibility of written medical information as the main barrier to adequate communication within the healthcare organization. Furthermore, patient perspectives emerged about the value of the collaboration between health sectors, as well as the perceived lack of accessibility and availability of healthcare services.

#### 3.6.1. Comprehensibility of Medical Documents and Information

The incomprehensibility of written medical information was reported to be caused by several different aspects: many patients with LHL criticized the use of medical jargon in the medical documents that made the information difficult to understand. Patients articulated that they preferred reliable information and even scientific evidence to be presented in a clear, concise, and understandable way and in plain language [45,46,58].

According to patients with LHL, the length and amount of text and the perceived difficulty of the text further prevented patients to read medical information to begin with [26,116]. The use of graphic illustrations and images are well received by patients as they ease readability and understanding for the patients [30,66]. Detrimental for comprehensibility is the fact that information from physicians and health information sources is not always coherent. A patient explains: “*There’s too much jargon (in health leaflets) they’re not written for lay people*” [45].

According to patients with LHL, they would benefit from clear, realistic and practical instructions or instruments on how to manage their disease or lifestyle as it results in positive behaviour change and made them feel like they can translate information into action [43,54,55,65,81,87,92,93,110,112,117]. In particular, hands-on demonstrations showing what to do was helpful according to patients with LHL, as well as receiving visual information [30,74].

Furthermore, patients with LHL criticized that the content covered in written medical information leaflets or handbooks is often repetitive and rarely novel [115]. This results in patients with LHL describing limited knowledge, experience or expectation prior to the diagnosis that affected them [64]. The majority of participants expressed a need for comprehensive information to be provided, as they had false beliefs of their condition, e.g., equating a cancer diagnosis to a ‘death sentence’ [115]. Fear of false expectations was described as affecting numerous areas of the prevention and care journey.

In contrast, videos helped increase patients’ involvement in conversations with their physicians about their disease and encouraged patients to raise questions to their physician. Important to the patients for the comprehensibility of the video material was an empathic, realistic, and appealing character, simple words and sentences, and step-wise changes in behaviour, and clear messages [46,117].

Information should be in line with patients’ needs and expectations and should not be too overwhelming [43,79]. Otherwise it results in confusion and feeling less confident.

#### 3.6.2. Availability and Accessibility of Health Care Providers

Patients reported that they perceive a high number of barriers to accessing healthcare caused by several different reasons: distance to healthcare facility, long waiting times, long waiting list for appointments, perceived lack of availability of timely outpatient care, and perceptions that the emergency care provides comprehensive care [43,75,102].

Some patients perceived that accessing care through an emergency care unit, leading to specialist care in the hospital would be a faster way to access care, as opposed to accessing it through primary care [75,102].

#### 3.6.3. Collaboration among Health Sectors and HCPs

Patients with LHL articulated the importance of involvement and alignment of all healthcare services (e.g., primary care, secondary care, pharmacies) in their prevention and care process. For example, it was helpful to patients when pharmacists provided further information and explanations on medications prescribed by their general practitioner (GP) and supported them in managing their medication regimes [43,102]. This facilitated their health knowledge and health literacy skills and provided them with information they could then discuss further with their GP. Patients also commented on the lack of communication between hospital clinics and their GP and described the lack of collaborative communication between primary and secondary care as an important barrier to effective disease management [102].

Patients expressed that they often saw different doctors, which required them having to repeatedly explain their medical history, resulting in low partnership between patients and doctors and less trust. Furthermore, patients experienced little communication surrounding transitions between primary and secondary care, resulting in many unanswered questions. A patient explains: “*…there has been no communication…between the…clinic and my GP…I can only tell him what I understand in the clinic*” [102]. 

### 3.7. Cultural Sensitivity

Cultural sensitivity emerged as a recurring theme throughout our analysis. Patients with LHL may come from either majority or minority populations, and cultural background seems to play a role in how health and the care process is perceived. Patients with LHL from minority cultural backgrounds, for example, expressed feeling more comfortable and confident when HCPs acknowledged and accommodated relevant cultural health beliefs and norms, family values and decision-making, and language. This was found across a variety of studies and sample sizes, with various populations including, for example, individuals of a Latin American background in the United States, various minority groups in Western European countries, and Aboriginals in Australia [22,24,28,29,32,33,39,46,58,85,88,90,92,94,104,107,119].

Repeatedly mentioned by patients with LHL from minority cultural backgrounds was the difference in expectations and understanding of treatment and chronic disease from their own background, e.g., the expectation that Western medicine provides instant healing. For example, some Cambodian patients were reluctant to use medication and therapies [85]. Cultural beliefs concerning treatment and any potential lack of understanding of chronic diseases can lead to a cycle of adherence and non-adherence. A patient explains: “*…If you take the herbals you must put the medicines aside. If you take the medicines, you must leave the herbals aside*” [85].

In addition, patients with LHL from non-Western cultures viewed disclosing something like, for example, a cancer diagnosis, to family and friends as a matter of shame, since a diagnosis of a chronic disease was associated with wrong-doing in the past [32]. Such cultural perceptions and beliefs may then be associated with a lack of support within the family or community that HCPs need to be aware of and take into account when defining the treatment plan for these patients.

In several studies, patients with LHL identified the importance of cultural norms and values related to the role of the family. For example, in cultures that are less individualistic than Western cultures, the role of family members in the care process of an individual patient was very important and could be a facilitator or a barrier for the patient [24].

While medical jargon and terminology used by HCPs was already mentioned by patients with LHL as a barrier to accessing, understanding, and using written medical information and the healthcare system, different languages may amplify this effect. Many patients who were not native speakers of the local language wished there were written information sources available in their own native language, or the presence of an interpreter during the consultations [29,58,101].

It is important for health professionals to acknowledge language and culture as potentially important factors for patients with LHL to understand and appraise information during the encounter.

### 3.8. eHealth

eHealth emerged as a recurring theme as the use of online tools and technology became repetitively apparent for patients’ care and treatment process. Online tools and technology seem to be used for several purposes and at different disease stages, for example, finding information about a symptom/disease, looking for support through online fora, using a self-management app for empowerment, or interacting and communicating with a medical provider via an online chatroom [34,55,80,89]. However, it was often mentioned by patients that eHealth tools are not sufficient in itself, but may rather serve as a supplemental tool to seek information, or as a means to ask additional questions to their physician, find peer support groups or fora, and use different self-management tools [53,57,120]. A patient explains: “*I have relied on the Internet in the sense of making contact with other patients with the same disease in the world”* [50].

Apart from the benefit of eHealth, patients with LHL reported barriers in using eHealth especially related to accessing and navigating the Internet. For example, they encountered difficulties with evaluating the relevance and reliability of various internet sources and they reported an overload of information they could find [50,122]. Furthermore, medical information was often difficult to understand and concerns about the security of the Internet were reported [57,67,80].

Due to this wide variety of patient experiences with and uses of eHealth, no subthemes within eHealth could be identified. Instead, it indicates its versatility and relevance for different themes and thus different care stages of patients.

#### Learning Outcomes

The second aim of the study was to enrich and adapt our educational framework and formulate learning outcomes. Table 4 displays an overview of relevant learning outcomes within each theme. All learning outcomes were formulated based on the findings within each theme. Person-centred care was identified as relevant for the patients’ whole care process and thus lays the foundation within the educational framework.

## 4. Discussion

This systematic review shows the relevance of person-centeredness in healthcare from the perspective of patients with limited health literacy. The synthesis of patient perspectives revealed four key domains that patients view as important for a person-centred care process: (1) Support system, (2) Patient self-management, (3) Interpersonal capacities of HCPs, (4) Barriers in healthcare systems, as well as two recurring themes: “cultural sensitivity” and “eHealth”. Our study confirms previous literature requesting a comprehensive HL educational program that takes into account perspectives from patients, HCPs, and researchers to improve future HCPs capacities and promote person-centred care [14,17,124,125]. Perspectives of patients provided valuable input to inform all domains of the educational framework, resulting in person-centred learning outcomes.

The key themes and sub themes that were identified from synthesizing patient perspectives in this systematic review could all be related to the educational framework but did not fully align. For example, the synthesis of patient perspectives revealed that additionally to communities, family, and peers, patients need HCPs to be part of their support system. Furthermore, sometimes the names of the themes were adapted from the domains of the educational framework to specifically fit the patients’ needs, preferences, and experiences. For example, the theme ‘patient self-management’ was adapted from the domain ‘empowering people with LHL’. Rather than to be empowered by their HCP, patients describe their needs and preferences in terms of tools that help them cope and self-manage their condition in their everyday lives. 

“Cultural sensitivity” and “eHealth” were identified as recurring themes being found in relation to all key themes. Cultural sensitivity may influence patients’ needs and preferences on all topics including diverse understanding of communicated information, non-verbal communication, cultural beliefs regarding health and care. It was previously established that LHL is a contributing factor for poor understanding and adherence, and limited access to health care in groups of minorities [126,127,128]. The combination of patient (cultural) diversity and health literacy is seen as a dual challenge as LHL is more prevalent in racial and ethnic minority populations and people with lower socioeconomic status causing greater health disparities [126,129]. For this reason, it is recommended for the development and creation of health profession curricula to stress the relevance and recognition of sensitivity to both health literacy and cultural diversity by creating cross-cultural education [127].

eHealth was found to be recurring throughout all themes as the (non-)use of eHealth tools and services was mentioned for multiple reasons, i.e., finding support networks and information, enhancing self-management, communicating online with HCPs, and navigating healthcare systems online. Although research has shown that persons with high levels of health literacy are more likely to adopt eHealth applications such as online personal health records than individuals with limited health literacy [130,131], our findings indicate that eHealth becomes increasingly important in the care process and is generally seen as a positive development from the perspective of patients with LHL. Previous research already revealed a strong association of HL with experiences searching for health information and preferences for health information sources [132]. For (future) healthcare providers it is, therefore, important to be aware of the needs, preferences, and abilities of people with LHL when facilitating the use of eHealth tools, for example when determining channels for health information dissemination or introducing self-management applications. 

As a next step, for every theme learning outcomes were formulated that capture the preferences, needs, and experiences of patients with LHL. Previous studies already suggested to develop a comprehensive, evidence-informed HL educational program to successfully improve future HCPs capacities [14,17,124,125]. Health literacy competencies and practices were formulated and prioritized based on the input of health literacy educators and experts in the United States using a Delphi method [17,125]. This study was replicated in a European setting applying a similar method [124]. A systematic review of learning outcomes for communication skills across the health professions [133] demonstrated that principles of person-centeredness are not consistently used as a basis for communication skills teaching. Only 5% of the included papers reported engaging patients in any aspect of the educational design [133]. To our knowledge, this is the first attempt to formulate learning objectives based on patient perspectives that need further confirmation in future studies.

The learning outcomes formulated in this systematic review are structured around the themes that were most significant for patients themselves. Several subthemes were identified pointing towards more specific topics that are to be addressed in educational programs related to HL and person-centred care. The majority of these subthemes could be gathered under similar themes as in the existing educational framework which confirms the validity of the original program but helps to formulate more specific learning outcomes that are truly person-centred, relevant to patients, and help to prioritize what (future) healthcare providers should learn. This list is complementary to the learning outcomes as formulated earlier by Coleman and colleagues [17] who provide a long list of learning outcomes to enhance HCPs knowledge, skills and attitudes in the area of health literacy. A next step would be to validate the learning outcomes from various studies with patients with LHL in a European setting.

This systematic review holds several strengths. Firstly, the review is unique in that it focuses entirely on the patient perspective. Secondly, our study benefits from the inclusion of a large number of articles and a wide range of information across a large set of chronic diseases. Thirdly, the involvement of different researchers in phase I and II of the data extraction and analysis in this review contributed to a high level of reliability and consequently of our findings. Finally, although we started with an educational framework, our analytic approach allowed us to stay close to the patients’ interpretations. We aimed to listen to the patient voice which led to patient-centred learning outcomes.

Despite these strengths, our study also had its limitations. Firstly, by using the explicit interpretation of the articles’ authors, we may possibly have missed important information that was not originally pointed out by the authors. Secondly, this study solely focused on individuals with LHL limiting the opportunities to compare this population with individuals holding various levels of health literacy. Thirdly, only a limited number of articles assessed health literacy with specific measurement tools which may have led to measurement errors with regard to level of health literacy. Fourthly, there could be some selection bias because a complete quality assessment of the articles was not performed. It was recommended to conduct a quality assessment [18,19,134] but there is little consensus on how to conduct methodological assessment of qualitative studies [19,134]. In this study, we aimed to include all patient voices and we believe that a complete quality assessment could have resulted in potentially important omissions. Fifthly, some patient perspectives might not have been included because we were unable to gain access to three articles. Lastly, although we focused fully on the patient perspective, when formulating the learning outcomes, we did not include them actively in this phase yet. For future research, we recommend validating these learning outcomes by consulting patients with LHL.

## 5. Conclusions

This systematic review confirms the relevance of training in person-centeredness and provides a conceptual model and learning outcomes that serve as input to build health literacy capacities of (future) HCPs in health care and education. A next step should be a participatory approach with patients, students and educators to develop a comprehensive educational program including further confirmation of the learning outcomes. 

## Figures and Tables

**Table 1:** Study characteristics.

Study Characteristics		*N*	%
*Countries*			
	USA	54	51.9
	UK	13	12.5
	Australia	14	13.5
	Canada	5	4.8
	Germany	4	3.8
	Netherlands	5	4.8
	Denmark	1	1.0
	Ireland	3	2.9
	Switzerland	1	1.0
	Sweden	1	1.0
	More than one country	3	2.9
*Qualitative methodology*			
	Interviews	63	61.2
	Focus groups	27	26.2
	Interviews and focus groups	7	6.8
*Type of chronic disease of the participants included in the study*			
	Diabetes	21	21.6
	Cancer	18	18.5
	Cardiovascular diseases (including stroke, hypercholesterolemia, hypertension, peripheral artery disease)	15	15.5
	Chronic kidney disease	8	8.2
	Musculosketal diseases (Arthritis/rheumatism; fibromyalgia; osteoporosis/osteopenia)	7	7.2
	HIV/AIDS	7	7.2
	Multiple chronic diseases within one study	7	7.2
	Gastroenterological diseases (Barrett’s columnar lined esophagus (CLO); Inflammatory bowel disease; Hepatitis B)	6	6.1
	Respiratory disease (Asthma/COPD)	6	6.1
	Other (chronic pain, disorders of the hematopoietic system)	4	4.1
	Mental health (depression, cognitive impairment)	2	2.1
	Urinary incontinence (overactive bladder symptoms, pelvic organ prolapse/urinary incontinence)	2	2.1

**Table 2:** Frequency of theme occurrence (*n* = 103 articles).

Theme	Frequency of Occurrence (*n*)
Support system	42
Patient self-management	60
Health care providers’ interpersonal capacities	62
Barriers in healthcare systems	34

**Table 3:** Summary of main results and quotations.

Themes and Sub-Themes		Example Quotations	Articles ^1^
*Support system*			
	Family and Friends	“It helps if you have someone eating along with you saying don’t eat this or don’t eat that. My sister encourages me to buy healthy food like I buy wheat noodles instead of regular noodles.” [22]	[22,23,24,25,26,27,28,29,30,31,32,33,34,35,36,37,38,39,40,41,42,43,44,45,46,47,48]
	Peer contact	“[..] They [other patients] can better inform you that as far as, versus a health provider that does not have the disease; they know how to treat the disease, but living with it is, is, you know, a different story.” [49]	[27,29,38,40,46,49,50,51,52,53,54,55,56,57,58]
	Religion and Spirituality	“So anyway, I went to the doctor […] we sat and we talked […] with all of the information that I received. Then I just thought. I said, “Listen, Alma, you’re a big girl, and these things happen.” I just began talking to myself, and I’m a believer in God. I just said I’m gonna put it in his hands. Whatever happens, whatever the diagnosis, if I have to have the mastectomy or whatever has to be done, I’m just gonna go ahead and have it done […] take your burden to the Lord and leave it there. I just really believe that God hears, and he answers prayers.” [25]	[25,32,40]
	Healthcare provider support	“I like the provider when he or she is concerned with me and the results and didn’t just turn their back and say “Oh, this is serious” and shut up.” [59]	[22,23,26,35,39,40,41,48,53,57,59,60,61,62]
*Patient self-management*			
	Autonomy and control	“It took me a long time to be the manager of my own health system. I expected doctors to kind of manage my life for me. It took me a long time to realize that no, I’m in charge of this. The doctors that work for me are a team, and I manage that team.” [63]	[25,36,41,48,54,60,64,65,66,67,68,69,70,71,72]
	Gaining knowledge	“The only way I’m able to cope is to have knowledge, which [doctors] think, if you don’t know, that’s how you’re going to be able to cope.” [73]	[22,25,30,31,34,35,37,38,44,46,48,52,54,55,56,71,73,74,75,76,77,78,79,80,81,82,83,84,85]
	Motivators	“The group leader has told us do it [control our blood sugar levels] for your [own] health and do it for your son. I don’t want my child to have this [diabetes]. I feel bad about having diabetes, having to take medications, worrying about what [to] eat . . . and sometimes get [ting] upset [because you don’t want your child to have diabetes]. I want to do whatever I can do to need less medication. We have to do our part.” [64]	[23,35,61,64,68,70,75,78,86,87,88,89,90,91,92,93]
	Monitoring	“… I know I’m working with someone that has access to my information and my dietary habits and what not, then that will mean that I’m going to try and stay within my dietary, good dietary, habits” [70]	[32,43,53,55,57,70,87,89]
*Healthcare providers’ interpersonal capacities*			
	Showing respect and understanding	“I didn’t feel like they were really interested. They were just talking… I just want my doctor to recognize who I am.. and they say: well let’s see how you doing.” [72] “The doctor understands me, devotes his time and listens to me. The patient also needs this aspect: to develop trust, to have a human relationship with the physician.” [60] “He [oncologist] knew me by my name, my face. When I came in, it was like they treated you like you were a person and not just cattle coming through. He used to call me his most delicate patient.” [65]	[22,23,28,30,31,42,48,50,52,57,59,60,61,62,65,68,72,76,77,87,88,90,91,94,95,96,97,98,99,100]
	Comprehensible communication	“[The doctor] was rattling off all these things that I needed to do…and my brain just shuts off. It was overload.” [91] “It’s the ability to explain in simple terms and not be abstruse. To say look, on the list here it says you’re to have 15 g of carbohydrates. Now that’s a slice of bread or that’s a medium sized potato.” [92] “I really didn’t pick up too much. I just feel that sometimes doctors go in and they’re using all these words and stuff. No. Tell me layman’s terms, because I’m not dumb, but something like that I don’t really understand.” [101]	[24,29,30,31,34,41,42,43,46,48,50,57,65,66,68,72,76,80,83,86,91,92,95,98,101,102,103,104,105,106,107,108,109]
	Enabling shared decision-making	“I know what each one of those pills are, what they’re for. I know that because I’m involved with my treatment, you know? Me and the doctor, we actually sit down and talk about me. I ask questions, you know? I’ve learned a lot. I mean, I’ve learned so much, really.” [76]	[34,37,38,43,56,60,62,65,66,70,73,76,79,83,91,100,101]
*Barriers in health care systems*			
	Comprehensibility of medical documents and information	“There’s too much jargon (in health leaflets) they’re not written for lay people”. [100]	[26,30,33,36,46,50,55,58,66,92,100,110,111,112,113,114,115,116,117]
	Availability and accessibility of healthcare providers	“[My] doctor would [not be able to see me at] ‘that particular time of day,’ so [I] just went on to emergency.” [75]	[31,33,36,47,52,61,75,87,88]
	Collaboration among health sectors and healthcare providers	“I had the pharmacist at the hospital phone me to double check I was having blood tests regularly and ask if everything had been explained to me and they double checked.” [43] “I think it would help more if we saw the same person every time, if possible. Because you go in there and you think, well, do they know all about me?” [90]	[23,43,62,68,87,90,92,102,118]
*Cultural sensitivity*			
		“…If you take the herbals you must put the medicines aside. If you take the medicines, you must leave the herbals aside.” [85] “I can say majority (sic) of the printed information is in English and the medical terminologies are very difficult for me to understand.” [58]	[22,24,28,29,32,33,39,46,58,85,88,90,92,94,104,107,119]
*eHealth*			
		“I have relied on the Internet in the sense of making contact with other patients with the same disease in the world” [50] “What I love…was that it told me how much to have and I didn’t overeat. The number of times that I overeat from hypos is ridiculous; it would be 99 % of times.” [120] “About the email, the one thing that I really like is that the doctor has always got somebody waiting for him, so the nurses are the ones that were logging in to the email and doing the routing of the-and letting him know what’s going on, what the-and I really like that ! That’s the sort of addresses the issue that you brought up.” [53]	[27,29,34,38,40,50,52,53,56,57,67,71,72,89,110,120,121,122]

^1^ Many articles discussed more than one theme. For every theme we included the references to the most significant articles related to this topic.

**Table 4:** Learning outcomes based on the perspectives of patients with chronic diseases and limited health literacy.

Themes	Learning Outcomes
*Key themes*	
Promoting person-centred care	Students should be able to… demonstrate a positive attitude towards person-centeredness and person-centred behaviours. … provide an overview of different communication goals and understand what these mean for the patient and the care process. … apply the concept of person-centred care and how to integrate this into practice.
Support system	Students should be able to… … explain the relevance, benefits, and potential negative impact of the involvement of family members, friends, peers and healthcare providers (support system) in the patient’s care process. … demonstrate the ability to identify the patient’s needs and preferences for the involvement of their support system. … demonstrate the ability to involve family members and friends based on individual patient’s needs and preferences. …recognize when a patient is facing a lack of support due to life circumstances. …reflect on their own support practices in order to improve these in patient care.
Patient self-management	Students should be able to… … provide patients with information (sources) that are understandable, reliable, relevant, accessible, and practically applicable. … appraise monitoring systems and positive accountability to promote patient-related outcomes (e.g., motivation, self-management skills).
Healthcare providers’ interpersonal capacities	Students should be able to… … demonstrate the ability to develop a positive provider-patient relationship based on mutual understanding and trust. … appraise the importance of comprehensible and comprehensive information. … translate medical information into easily and understandable information for patients to help them explore preferences and make decisions. … demonstrate the ability to use patients’ needs and preferences as the frame of reference for information exchange and making healthcare decisions. … recognize the influence of discontinuity of care at patient level.
Barriers in health care systems	Students should be able to… … explain the relevance of comprehensible written information for patients’ knowledge, motivation, participation and other outcomes. … understand the added value of information material such as images and videos to increase patient understanding and include this in their consultations. … initiate and support developments to enhance the comprehensibility of information provision in their health organization. … judge the importance to collaborate and communicate with fellow providers to ensure continuity of care. … demonstrate the ability to support patients struggling with the healthcare system and assist them in navigating it.
*Recurring themes*	
Cultural sensitivity	Students should be able to… … understand cultural health beliefs and norms, family values and decision-making, and language, … apply their knowledge of cultural aspects in the care process to by tailoring their communication to patients’ needs and preferences. … be aware of the beliefs and norms of the most prominent cultures/ethnicities in their work area. … communicate in a culturally sensitive way taking into account patients’ needs and preferences, e.g., by providing written information sources in their own native language, or facilitate the presence of an interpreter during the consultations.
eHealth	Students should be able to… … value the benefits of eHealth as an additional source of information and support. … facilitate the use of eHealth and integrate this in prevention and care. … provide patients with relevant information about eHealth tools beneficial for their specific care process. … value the latest developments on eHealth.
